# Imaginal retraining decreases craving for high-calorie food in overweight and obese women: A randomized controlled trial

**DOI:** 10.1038/s41398-019-0655-7

**Published:** 2019-11-28

**Authors:** Steffen Moritz, Anja S. Göritz, Stella Schmotz, Roland Weierstall-Pust, Josefine Gehlenborg, Jürgen Gallinat, Simone Kühn

**Affiliations:** 1 Department of Psychiatry and Psychotherapy, University Medical Center Hamburg-Eppendorf, Hamburg, Germany; 2 Department of Occupational and Consumer Psychology, University of Freiburg, Freiburg, Germany; 3 MSH Medical School Hamburg, University of Applied Sciences and Medical University, Hamburg, Germany

**Keywords:** Addiction, Psychiatric disorders

## Abstract

Overweight and obesity are epidemic conditions. Obesity is associated with somatic and psychological sequelae, including serious life-shortening disorders (e.g., diabetes). This study aimed to evaluate the efficacy of a newly developed imaginal variant of approach bias modification (i.e., imaginal retraining) for the reduction of craving for high-calorie food. In a randomized controlled trial, 384 women with a body mass index above 25 were allocated to a wait-list control group or to two variants of imaginal retraining (ratio: 1; 0.5; 0.5). The two intervention groups were sent a manual on imaginal retraining. One group was explicitly encouraged and instructed to use electronic reminders (R_ER_); the standard retraining group (R_S_) was not encouraged to use electronic reminders. Assessments were 6 weeks apart and were carried out online. Craving for high-calorie food represented the primary outcome (based on the Visual Analog Scale, VAS). Secondary outcomes included the Food Cravings Questionnaire (FCQ-T-R). The study was registered as DRKS00017220. Women in the R_ER_ group utilized the retraining technique more often than those in the R_S_ condition, and utilization frequency in turn was associated with improvement on craving and eating behavior scales. Both intention-to-treat and per-protocol analyses showed a favorable effect of the R_ER_ group, which achieved significance on the primary outcome, as well as on several other outcomes relative to controls at a small to medium effect size. For those participants who measured their weight before and after the assessment using a scale, weight loss in the R_ER_ group was significantly greater compared to the control group. Both retraining groups (R_ER_: 39.4%; R_S_: 31.1%) reduced their subjective amount of eating relative to controls (24.2%). Approximately two-thirds of the sample (68.3%) performed the exercises at least once during the study period. The present results show that, when used regularly, imaginal retraining may reduce craving for high-calorie food in overweight and obese women. Of note, there was also evidence suggestive of weight reduction, although no diet or lifestyle change was recommended in the manual. Because a large subgroup neither read the manual nor performed the exercises, we recommend that future imaginal retraining be conveyed via short video clips.

## Introduction

Overweight and obesity are growing problems with epidemic dimensions^1,2^. In Europe, 30.5% of women and 44.7% of men are considered obese, and 53.1% meet the criteria for being overweight^3^. Prevalence is especially high in industrialized countries, but the problem is also on the rise in developing countries^1,4^. Obesity is a primary risk factor for several disorders (e.g., cardiovascular disease, coronary heart disease, sleep apnea, atrial fibrillation, diabetes) and aggravates known risk factors (e.g., high blood pressure) for other life-shortening disorders (e.g., stroke). High BMI is regarded as responsible for 4 million deaths worldwide per year, representing 7.1% of deaths due to any cause and contributing to 120 million disability-adjusted life years^4^. Moreover, obesity is associated with psychological sequelae such as (self-)stigma^5^.

Standard measures to decrease weight are dieting (i.e., reducing the intake of calories) and/or increased physical activity to burn additional calories. In severe cases, surgery is recommended^6^ as a last resort despite possible side effects^7^. Some pharmacological agents have also shown promise^8^.

The present article is concerned with another route of intervention—the reduction of craving for food^1^ —by means of *approach bias modification* (ABM). ABM procedures are rooted in dual-process theories^9,10^, which try to explain substance and behavioral addictions, including obesity, as an excess of (implicit) impulsive approach tendencies that override both the (explicit) will to disengage from the substance or behavior and the insight into the negative consequences of the substance or behavior^11^. In line with the tenets of dual-process theories, experimental studies using the approach-avoidance task (AAT) show that individuals with addictive behavior are faster to pull a picture of their preferred/craved substance/object (e.g., drug, alcoholic drink, high-calorie food) toward themselves than to push it away via a joystick in a computerized set-up compared to non-addicted individuals^12^. This effect is well-established across a range of disorders, particularly substance disorders but also obesity (see ref. ^13^ for a study with an approach-avoidance variant of the implicit-association task), and speaks to an embodiment of addiction (embodiment or embodied cognition is the theory that many features of an agent’s cognition are shaped by aspects of his or her body^14^). Building upon diagnostic findings from the AAT and other assessment procedures, ABM aims to revert and decrease embodiment. The feasibility and effectiveness of the approach is well established in problem drinkers^15^ but has been successfully applied to other addictions as well^16^. In ABM, the addictive substance or behavior is coupled with pushing a joystick, which causes the picture to shrink in size, thus creating the illusion of distance, whereas a positive or neutral cue is coupled with pulling a joystick, which causes the picture to increase in size, thus creating the illusion of approach. Thus, ABM adopts the rationale of the AAT to decrease the approach bias towards addiction-related stimuli. The reduction of relapse in alcohol use disorder is usually small through ABM. Several studies have shown that ABM reduces the exaggerated approach bias in people with addictive behavior, particularly relating to alcohol^16–18^. The approach was then transposed to other addictions, particularly behavioral ones. For eating disorders results are mixed. In one meta-analysis, cognitive bias modification interventions that included ABM resulted in a medium change in the approach bias and small effects on food consumption and cravings for high-calorie food^19^. The latest meta-analysis found no effect of ABM on eating behavior. Likewise, a recent study, not included in the meta-analysis, found positive effects of ABM on the approach-avoidance bias but not on eating pathology questionnaires or the body mass index (BMI)^20^.

Recent meta-analyses, however, have questioned the general effectiveness of the ABM approach in ameliorating clinical outcomes (addiction and craving)^21,22^. In the meta-analysis by Boffo et al. ^22^, cognitive bias modification exerted a small effect on cognitive biases and relapse but not on the reduction of substance use.

The ABM procedure is simple to use, but even its developers acknowledge that the task can be boring and tedious for some individuals^23^. Another obstacle for routine implementation is the need for access to a computer device and a joystick. Moreover, when using computerized devices the available images are limited and difficult to personalize, particularly relating to the typical environment of intake^24^. Further, the many possible combinations of the substance that is craved (e.g., alcohol: wine, beer), brand/type of packaging, and favorite method of consumption (e.g., can, glass, bottle) are hard to capture with pictures.

Imaginal retraining aims to address these limitations. The technique consists of two phases that are easy for the participants to execute. Following a simple negative mood induction to enhance embodiment (for details, see the “Methods” section), the alleged primary mechanism of ABM^25^, in the averse sequence participants throw (the behavior/movement is actually performed) the *imagined* high-calorie food away from them. This sequence corresponds to the push movement in conventional ABM. For the opposite sequence, the user engages in a positive mood induction (see the “Methods” section for details) before imagining eating or drinking a delicious but healthy, low-calorie beverage, or food, while coupling this with other positive sensations to enhance the effects of embodiment^25^. This sequence corresponds to the pull movement in conventional ABM. Two randomized-controlled trials with problem drinkers^24^ and smokers^26^ showed that imaginal retraining reduced both craving for alcohol/cigarettes (proximal outcome), as well as actual drinking and smoking behavior (distal/clinical outcome).

For the first time, we examined the efficacy of imaginal retraining to reduce craving for high-calorie food (primary outcome) in individuals with overweight (BMI > 25). Secondary outcomes were the Three-Factor Eating Questionnaire (part of the German Fragebogen zum Essverhalten, FEV; German version^27^); the Food Cravings Questionnaire-Trait-reduced (FCQ-T-R)^28^, well-being (WHO Quality of Life scale)^29^, and weight. We adapted the original manual, which we had developed for problem drinkers and smokers, to people with overweight and obesity. The new manual encouraged neither dieting nor any additional physical activity. The (wait-list) control group received access to treatment upon completion of the post assessment.

## Methods

The web-based intervention was conducted via WisoPanel^30,31^, a participant pool of German-speaking individuals who have registered for participation in web-based studies (https://www.wisopanel.net). Members of WisoPanel are drawn from diverse sources comprised of people from all social classes and is demographically similar to the general population.

**Fig. 1:**
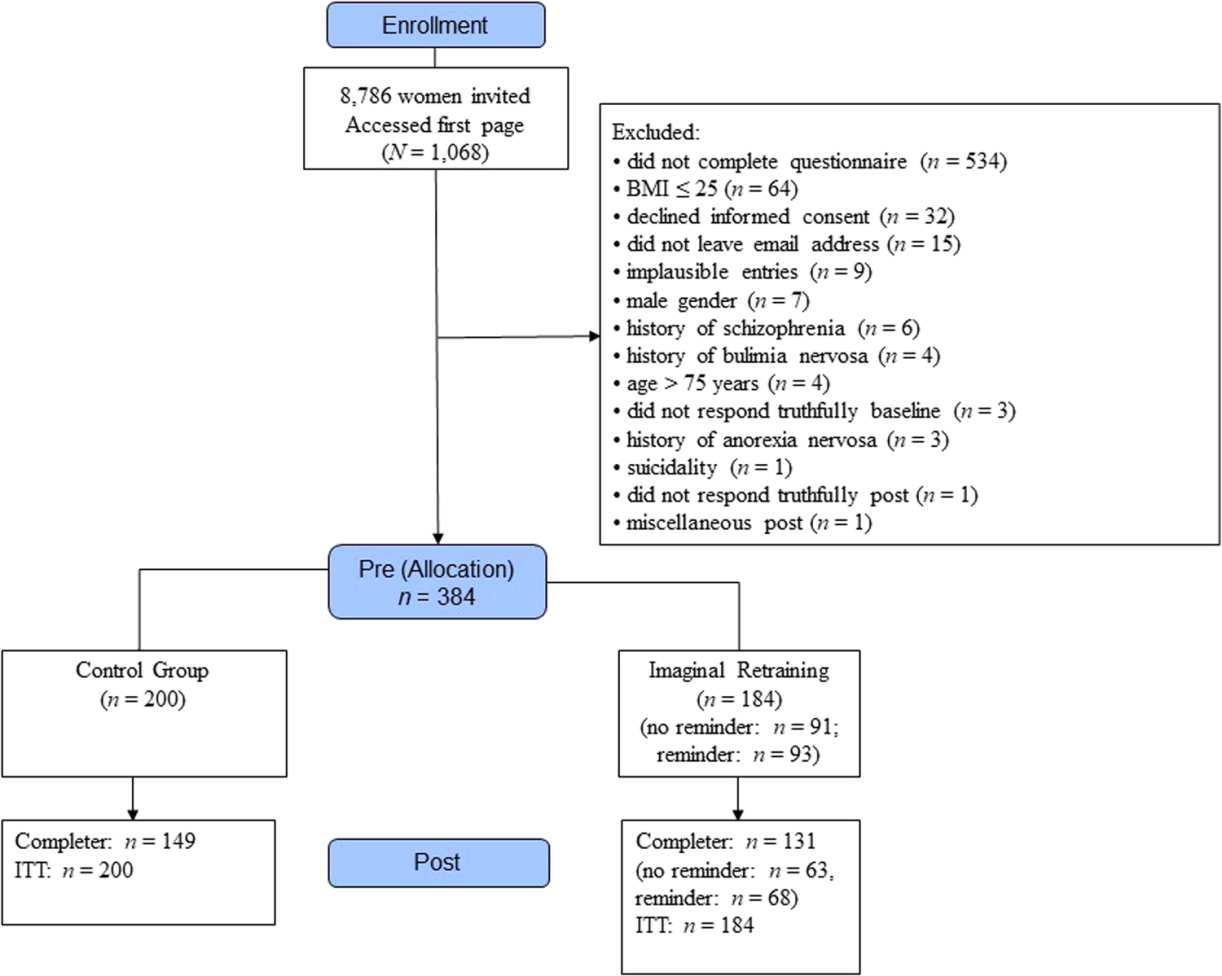
CONSORT flow chart for the current study. ITT = intention-to-treat analysis.

Because the BMI of women registered with WisoPanel was not known and this was the primary inclusion criterion of the study, all registered women (*N* = 8.786 at the time of inclusion) received an invitation and a link to the study. The invited sample thus was comprised of women who were normal weight, overweight, or obese. After a short description of the purpose of the study, interested people were asked to give their explicit informed consent if they met the inclusion criteria (see below). Inclusion criteria were age between 18 and 75 years and a BMI > 25 (we calculated this based on the weight and height of the participants). A history of anorexia, bulimia, or psychosis led to exclusion. Acute suicidality (as manifested by a score of 2 or 3 on the BDI-II rating for suicidal ideation) led to automatic exclusion with a referral to specific websites offering help, including emergency numbers (see Fig. 1).

### Design

The intervention was unguided; the study was set up as a randomized-controlled trial (randomization plan); controls were allowed access to standard care. Study participation was anonymous; participants were advised to create email addresses that did not disclose their names. Participants in the intervention condition received the imaginal retraining manual immediately after randomization via an email attachment, whereas participants in the control condition received the manual upon completion of the post assessment. One week after the initial sending of the manual, half of the intervention sample received an email with specific instructions on how to set the timer on their smartphone in order to receive daily reminders to perform the exercises (no participant canceled the study nor was excluded from it for not having a smartphone or not being willing to use it).

Data from 384 participants were included in the final analyses (see Table 1), which according to g*power allowed for the detection of a small effect size. The main reasons for exclusion of data were cancellation of the survey before completion, BMI ≤ 25, and refusal to give informed consent (see CONSORT flow chart in Fig. 1). The trial was registered with the German Clinical Trials Register (DRKS0001722). We carefully checked that required statistical assumptions were met (e.g., normal distribution; equal variances—for certain comparisons adjustments for unequal variances were made).

### Invitation and baseline survey

Assessments were conducted online using Questback/UniPark®. In keeping with guidelines of the European General Data Protection Regulation (GDPR), no IP addresses were stored. The local ethics committee for psychologists at the University Hospital of Hamburg-Eppendorf (Germany) approved the study (LPEK-0030). As an incentive for participation, a self-help manual on imagery rescripting was sent to all individuals who completed the study^32^, as well as a link to the MCT & More app (www.uke.de/mct_app), which is aimed to improve self-esteem.

At the start of the survey, electronic informed consent was required. Questions on the participant’s sociodemographic background as well as medical history (e.g., prior experience with psychotherapy; current treatment status; prior psychiatric diagnoses, if any) followed. Subsequently, scales on eating behavior and well-being were presented (see section “Questionnaires”). We also asked for the participant’s present weight. At the end, we asked participants whether they had truthfully answered the questions (inclusion criterion). We requested a pseudonymized email address. Email addresses were not stored online. Within 24 h, participants were randomized to one of the three conditions (ratio: 1 (wait list); 0.5 standard retraining; 0.5 retraining with electronic reminders). The allocation was implemented based on the time the participant entered the study. Owing to the design of the online study, which involved no personal contact, concealed allocation as in clinical trials with face-to-face assessments could not be implemented. Our procedure is best characterized as centralized assignment with no risk of bias because the person allocating individuals to conditions had no information about participants other than the date they had signed up for the study and their anonymized email.

Participants in the control group were informed via email that they would receive the retraining manual after the post assessment. Six weeks after initial participation, all participants were invited to participate in the post assessment. Up to three reminders were sent. For the post assessment, participants were first asked to re-enter their email address to allow matching of pre and post data. The same set of questionnaires was administered as in the baseline survey. We asked participants for their present weight and whether this was measured with a scale (this question was entered during the follow-up period and the results are therefore available for a subgroup; see Tables 2 and 3). Further, we asked whether they had eaten less during the study period vs. the same or more as previously (the latter two categories were later pooled). Those in the retraining conditions who had at least started to read the manual were asked further questions related to their subjective assessment of the quality, comprehensibility, satisfaction, and efficacy of the manual (see Tables 4 and 5).

**Table 1:** Demographics, well-being, and eating-related characteristics of the sample at baseline.

	Wait-list control (C; *n* = 200)	Retraining standard (R_S_; *n* = 91)	Retraining electronic reminders (R_ER_, *n* = 93)	Statistics (df: 2, 381)
*Baseline characteristics*				
Age in years	48.79 (11.77)	49.35 (9.89)	47.77 (11.61)	*F* = 0.47, *p* = 0.627
Diets in parallel in %	21.5%	20.6%	22.1%	*χ*^2^ (1) = 0.04, *p* = 0.980
Height in cm	166.63 (6.71)	165.71 (6.62)	166.73 (7.21)	*F* = 0.68, *p* = 0.508
Weight in kg	90.34 (17.31)	89.37 (18.56)	88.79 (18.70)	*F* = 0.26, *p* = 0.769
BMI	32.56 (6.13)	32.46 (5.99)	31.91 (6.15)	*F* *=* 0.38, *p* = 0.687
Obesity (BMI > 25 < 30; BMI ≥ 30 < 35; BMI ≥ 35) in %	42.0/30.0/28.0%	39.5/34.1/26.4%	51.6/23.7/24.7%	*χ*^2^ (4) = 3.82, *p* = 0.431
*Eating scales*				
VAS	6.74 (2.42)	6.45 (2.52)	6.60 (2.35)	*F* *=* 0.48, *p* = 0.619
FCQ-T-R				
Lack of control	16.56 (4.79)	16.47 (5.41)	16.71 (5.10)	*F* *=* 0.05, *p* = 0.648
Thoughts/preoccupation with food	13.65 (5.61)	12.98 (5.49)	13.44 (6.11)	*F* *=* 0.43, *p* = 0.649
Intentions to consume	6.41 (2.16)	6.30 (2.27)	6.38 (2.22)	*F* *=* 0.08, *p* = 0.927
Emotions	6.75 (2.39)	6.63 (2.62)	6.95 (2.34)	*F* *=* 0.41, *p* = 0.664
Triggers	3.77 (1.09)	3.52 (1.28)	3.61 (1.17)	*F* *=* 1.59, *p* = 0.206
Total	47.13 (13.74)	45.89 (14.85)	47.09 (14.31)	*F* *=* 0.26, *p* = 0.770
FEV cognitive restraint	8.39 (4.16)	7.93 (4.26)	7.70 (4.27)	*F* = 0.95, *p* = 0.388
FEV disinhibition	9.72 (3.49)	8.90 (4.26)	9.78 (3.75)	*F* = 1.75, *p* = 0.175
FEV hunger	7.66 (3.62)	7.05 (3.68)	7.25 (3.45)	*F* = 1.00, *p* = 0.367
*Psychological scales*				
BDI	14.54 (10.49)	11.78 (8.64)	15.72 (9.24)	*F* *=* 4.02, *p* = 0.019 (R_ER_ > R_S_: *p* = 0.007), R_S_ < C: *p* = 0.026)
WHOQOL	3.43 (0.87)	3.63 (0.83)	3.45 (0.79)	*F* *=* 1.86, *p* = 0.157
expectancy	5.48 (1.73)	5.19 (2.00)	5.51 (1.61)	*F* = 1.01, *p* = 0.365

Means and standard deviations (in brackets)

*BDI-II* Beck Depression Inventory II, *BMI* body mass index, *FCQ-T-R* Food Cravings Questionnaire-Trait-reduced, *FEV* Three-Factor Eating Questionnaire (part of the Fragebogen zum Essverhalten, FEV), *VAS* Visual Analog Scale (craving for high-calorie food), *WHOQOL-BREF* WHO Quality of Life

**Table 2:** Intention-to-treat analyses for the pooled retraining group and subgroup with electronic reminders (R_ER_) vs. controls.

	Wait-list controls		Retraining standard (R_S_)		Retraining with reminders (R_ER_)		Mixed ANOVA (df = 1229)
	Pre	Post	Pre	Post	Pre	Post	WLC vs. Retraining overall [C vs. R_ER_]
VAS	6.56 (2.56)	6.21 (2.04)*	6.57 (2.49)	6.19 (2.02) [n.s.]	6.76 (2.30)	5.60 (1.79)****	Time: *F* *=* 23.37, *p* < 0.001, *η*_p_^2^ = 0.067 Interaction: *F* *=* 0.95, *p* = 0.329, *η*_p_^2^ = 0.002 [C < R_ER_: *p* = 0.011, *η*_p_^2^ = 0.022]
FCQ-T-R							
Lack of control	16.25 (4.92)	15.79 (5.25) [n.s.]	15.89 (5.44)	14.44 (5.21)***	17.15 (4.80)	15.04 (4.88)****	Time: *F* *=* 41.10, *p* < 0.001, *η*_p_^2^ = 0.097 Interaction: *F* *=* 11.57, *p* = 0.001, *η*_p_^2^ = 0.029 [C < R_ER_: *p* = 0.002, *η*_p_^2^ = 0.032]
Thoughts/preoccupation with food	13.42 (5.63)	13.00 (5.89) [n.s.]	12.29 (5.42)	12.08 (5.03) [n.s.]	13.65 (6.06)	12.24 (5.53)*	Time: *F* *=* 12.45, *p* < 0.001, *η*_p_^2^ = 0.032 Interaction: *F* *=* 1.13, *p* = 0.288, *η*_p_^2^ = 0.003 [C < R_ER_: *p* = 0.039, *η*_p_^2^ = 0.015]
Intentions to consume	6.30 (2.22)	6.07 (2.32) [n.s.]	6.19 (2.24)	5.67 (2.09)*	6.51 (2.15)	5.81 (2.10)*	Time: *F* *=* 18.49, *p* < 0.001, *η*_p_^2^ = 0.046 Interaction: *F* *=* 2.73, *p* = 0.100, *η*_p_^2^ = 0.007 [C = R_ER_: *p* = 0.068, *η*_p_^2^ = 0.011]
Emotions	6.79 (2.35)	6.47 (2.52)*	6.37 (2.60)	6.17 (2.33) [n.s.]	7.10 (2.25)	6.43 (2.33)***	Time: *F* *=* 20.97, *p* < 0.001, *η*_p_^2^ = 0.052 Interaction: *F* *=* 1.85, *p* = 0.174, *η*_p_^2^ = 0.005 [C = R_ER_: *p* = 0.107, *η*_p_^2^ = 0.009]
Triggers	3.73 (1.08)	3.46 (1.13)****	5.51 (1.27)	3.14 (1.24)*	3.66 (1.14)	3.24 (1.15)***	Time: *F* *=* 45.10, *p* < 0.001, *η*_p_^2^ = 0.106 Interaction: *F* *=* 6.95, *p* = 0.009, *η*_p_^2^ = 0.018 [C = R_ER_: *p* = 0.141, *η*_p_^2^ = 0.007]
Total	46.49 (14.01)	44.79 (15.01)*	44.24 (14.95)	41.51 (13.92)*	48.07 (13.27)	42.75 (13.66)****	Time: *F* *=* 36.30, *p* < 0.001, *η*_p_^2^ = 0.087 Interaction: *F* = 5.20, *p* = 0.023, *η*_p_^2^ = 0.013 [C < R_ER_: *p* = 0.007, *η*_p_^2^ = 0.025]
FEV							
Cognitive restraint	8.65 (4.05)	8.90 (4.50) [n.s.]	8.27 (4.55)	9.11 (4.84)^+^	7.97 (4.31)	9.29 (4.48)****	Time: *F* *=* 27.17, *p* < 0.001, *η*_p_^2^ = 0.066 Interaction: *F* *=* 6.95, *p* = 0.009, *η*_p_^2^ = 0.018 [C < R_ER_: *p* = 0.006, *η*_p_^2^ = 0.026]
Disinhibition	9.70 (3.36)	8.87 (3.67)****	8.73 (4.49)	7.92 (3.79)*	10.09 (3.63)	8.29 (3.89)****	Time: *F* *=* 56.10, *p* < 0.001, *η*_p_^2^ = 0.128 Interaction: *F* *=* 2.32, *p* = 0.128, *η*_p_^2^ = 0.006 [C < R_ER_: *p* = 0.005, *η*_p_^2^ = 0.026]
Hunger	7.58 (3.71)	7.27 (3.79) [n.s.]	6.70 (3.77)	5.94 (3.63)*	7.46 (3.31)	6.51 (3.56)*	Time: *F* *=* 16.86, *p* < 0.001, *η*_p_^2^ = 0.042 Interaction: *F* *=* 4.21, *p* = 0.041, *η*_p_^2^ = 0.011 [C = R_ER_: *p* = 0.074, *η*_p_^2^ = 0.011]
BDI	14.69 (10.53)	12.54 (10.70)****	11.92 (8.42)	10.21 (8.75)*	16.12 (8.63)	12.34 (9.40)****	Time: *F* *=* 69.43, *p* < 0.001, *η*_p_^2^ = 0.154 Interaction: *F* *=* 1.06, *p* = 0.305, *η*_p_^2^ = 0.003 [C < R_ER_: *p* = 0.017, *η*_p_^2^ = 0.019]
WHO QoL	3.41 (0.90)	3.48 (0.96) [n.s.]	3.65 (0.83)	3.52 (0.72) [n.s.]	3.50 (0.80)	3.68 (0.74)*	Time: *F* *=* 2.70, *p* = 0.101, *η*_p_^2^ = 0.007 Interaction: *F* *=* 0.02, *p* = 0.898, *η*_p_^2^ < 0.001 [C = R_ER_: *p* = 0.081, *η*_p_^2^ = 0.010]
Weight in kg (control: *n* = 39; R_S_: *n* = 12; R_ER_: *n* = 23)	94.89 (20.36) (17.43)	94.48 (20.81) [n.s.]	98.12 (23.15)	97.57 (22.74) [n.s.]	82.03 (19.93)	78.53 (12.37)*	Time: *F* *=* 5.16, *p* = 0.026, *η*_p_^2^ = 0.068 Interaction: *F* *=* 2.93, *p* = 0.060, *η*_p_^2^ = 0.076 [C < R_ER_: *p* = 0.033, *η*_p_^2^ = 0.074]

During the study period, we added two questions on present weight and whether weight was measured via a scale at both assessment points. Therefore, this variable is only available for a subgroup of individuals

*BDI-II* Beck Depression Inventory II, *BMI* body mass index, *FCQ-T-R* Food Cravings Questionnaire-Trait-reduced, *FEV* Three-Factor Eating Questionnaire (part of the Fragebogen zum Essverhalten, FEV), *VAS* Visual Analog Scale (craving for high-calorie food), *WHOQOL-BREF* WHO Quality of Life

n.s. = non-significant; ^+^*p* *<* 0.1; *p *<* 0.05; **p *<* 0.01; ***p *<* 0.005; ****p *<* 0.001

**Table 3:** Per-protocol comparisons (technique executed at least once during intervention period) for pooled retraining group and subgroup with electronic reminders (R_ER_) vs. controls.

Variable	Wait-list controls		Retraining pooled (*n* = 82)		Retraining with reminders (R_ER_) (*n* = 44)		Mixed ANOVA (df = 1229)
	Pre	Post	Pre	Post	Pre	Post	WLC vs. retraining overall [C vs. R_ER_]
VAS	6.56 (2.56)	6.21 (2.04)*	7.11 (1.99)	6.03 (1.68)****	7.35 (1.80)	5.86 (1.49)****	Time: *F* = 22.64, *p* < 0.001, *η*_p_^2^ = 0.090 Interaction: *F* = 5.83, *p* = 0.010, *η*_p_^2^ = 0.025 [C < R_ER_: *p* = 0.002, *η*_p_^2^ = 0.051]
FCQ-T-R							
Lack of control	16.25 (4.92)	15.79 (5.25) [n.s.]	17.61 (4.89)	15.07 (4.79)****	18.66 (4.26)	15.73 (4.66)****	Time: *F* *=* 27.68, *p* < 0.001, *η*_p_^2^ = 0.108 Interaction: *F* = 13.30, *p* < 0.001, *η*_p_^2^ = 0.055 [C < R_ER_: *p* = 0.001, *η*_p_^2^ = 0.059]
Thoughts/preoccupation with food	13.42 (5.63)	13.00 (5.89) [n.s.]	13.50 (5.93)	12.35 (5.30)*	14.45 (6.38)	12.55 (5.68)*	Time: *F* = 7.40, *p* = 0.007, *η*_p_^2^ = 0.031 Interaction: *F* = 1.60, *p* = 0.207, *η*_p_^2^ = 0.007 [C < R_ER_: *p* = 0.040, *η*_p_^2^ = 0.022]
Intentions to consume	6.30 (2.22)	6.07 (2.32) [n.s.]	6.73 (2.10)	5.73 (1.92)****	6.80 (2.06)	5.86 (2.03)**	Time: *F* *=* 19.64, *p* < 0.001, *η*_p_^2^ = 0.079 Interaction: *F* = 7.49, *p* = 0.007, *η*_p_^2^ = 0.032 [C < R_ER_: *p* = 0.048, *η*_p_^2^ = 0.020]
Emotions	6.79 (2.35)	6.47 (2.52)*	7.04 (2.45)	6.49 (2.21)**	7.50 (2.25)	6.57 (2.26)****	Time: *F* = 10.85, *p* = 0.001, *η*_p_^2^ = 0.045 Interaction: *F* *=* 0.77, *p* = 0.380, *η*_p_^2^ = 0.003 [C = R_ER_: *p* = 0.061, *η*_p_^2^ = 0.018]
Triggers	3.73 (1.08)	3.46 (1.13)****	3.73 (1.11)	3.17 (1.12)****	3.68 (1.12)	3.16 (1.06)***	Time: *F* *=* 35.31, *p* < 0.001, *η*_p_^2^ = 0.134 Interaction: *F* *=* 4.32, *p* = 0.039, *η*_p_^2^ = 0.019 [C = R_ER_: *p* = 0.145, *η*_p_^2^ = 0.011]
Total	46.49 (14.01)	44.79 (15.01)*	48.61 (13.47)	42.82 (12.95)****	51.09 (12.75)	43.86 (13.35)****	Time: *F* = 24.68, *p* < 0.001, *η*_p_^2^ = 0.098 Interaction: *F* = 7.35, *p* = 0.007, *η*_p_^2^ = 0.031 [C < R_ER_: *p* = 0.004, *η*_p_^2^ = 0.043]
FEV							
Cognitive restraint	8.65 (4.05)	8.90 (4.50) [n.s.]	8.57 (4.52)	10.13 (4.49)****	8.30 (4.32)	10.00 (4.03)****	Time: *F* *=* 15.91, *p* < 0.001, *η*_p_^2^ = 0.065 Interaction: *F* *=* 8.34, *p* = 0.004, *η*_p_^2^ = 0.035 [C < R_ER_: *p* = 0.009, *η*_p_^2^ = 0.035]
Disinhibition	9.70 (3.36)	8.87 (3.67)****	9.91 (4.02)	8.37 (3.66)****	11.11 (3.17)	8.93 (3.75)****	Time: *F* *=* 36.24, *p* < 0.001, *η*_p_^2^ = 0.137 Interaction: *F* *=* 3.38, *p* = 0.067, *η*_p_^2^ = 0.015 [C < R_ER_: *p* = 0.004, *η*_p_^2^ = 0.042]
Hunger	7.58 (3.71)	7.27 (3.79) [n.s.]	7.28 (3.24)	6.04 (3.22)****	7.77 (3.12)	6.48 (3.44)**	Time: *F* = 14.55, *p* < 0.001, *η*_p_^2^ = 0.060 Interaction: *F* = 5.24, *p* = 0.023, *η*_p_^2^ = 0.022 [*C* = R_ER_: *p* = 0.055, *η*_p_^2^ = 0.019]
BDI	14.69 (10.53)	12.54 (10.70)****	13.91 (8.05)	10.65 (8.59)****	16.73 (7.98)	12.84 (9.14)****	Time: *F* *=* 38.36, *p* < .001, *η*_p_^2^ = 0.145 Interaction: *F* = 1.66, *p* = 0.199, *η*_p_^2^ = 0.007 [C = R_ER_: *p* = 0.102, *η*_p_^2^ = 0.014]
WHO QoL	3.41 (0.90)	3.48 (0.96) [n.s.]	3.63 (0.84)	3.66 (0.71) [n.s.]	3.52 (0.73)	3.75 (0.69)*	Time: *F* *=* 0.79, *p* = 0.374, *η*_p_^2^ = 0.003 Interaction: *F* = 0.20, *p* = 0.651, *η*_p_^2^ = 0.001[C = R_ER_: *p* = 0.255, *η*_p_^2^ = 0.007)
Weight verified (wait-list: *n* = 39; pooled: *n* = 24, R_ER:_ *n* = 18)	94.89 (20.36	94.48 (20.81 [n.s.]	88.88 (21.33)	86.39 (20.78)^+^	83.48 (15.28)	80.16 (12.83)^+^	Time: *F* *=* 5.52, *p* = 0.022, *η*_p_^2^ = 0.083 Interaction: *F* = 2.87, *p* = 0.096, *η*_p_^2^ = 0.045 [C < R_ER_: *p* = 0.041, *η*_p_^2^ = 0.074]

During the study period, we added two questions on present weight and whether weight was measured via a scale at both assessment points. Therefore, this variable is only available for a subgroup of individuals

*BDI-II* Beck Depression Inventory II, *BMI* body mass index, *FCQ-T-R* Food Cravings Questionnaire-Trait-reduced, *FEV* Three-Factor Eating Questionnaire (part of the Fragebogen zum Essverhalten, FEV), *VAS* Visual Analog Scale (Craving for high-calorie food), *WHOQOL-BREF* WHO Quality of Life

n.s. = non-significant; ^+^*p* < 0.1; *p < 0.05; **p *<* 0.01; ***p < 0.005; ****p *<* 0.001

**Table 4:** Subjective appraisal by users of imaginal retraining (adapted from the Patient Satisfaction Questionnaire, German acronym ZUF-8; Schmidt et al. 1989), with percentage of positive vs. negative appraisals, means and standard deviations (results of the imaginal retraining trial in problem drinkers are inside square brackets).

Item	Percentage of positive vs. negative appraisals; means		Statistics
	Imaginal retraining standard (R_S_; *n* = 38)	Imaginal retraining with reminders (R_ER_; *n* = 44)	
How do you rate the quality of the manual? (excellent (1), good (2) vs. less good (3), not good (4))^a^	81.1% (2.21 (0.74))	92.% (2.18 (1.06))	*χ*^2^(1) = 2.22, *p* = 0.136 [81.8%] [2.05 (0.58)]
Did you receive the type of treatment you expected to receive? (not at all (1), not really (2) vs. in general, yes (3), yes, absolutely (4))	61.1% (2.82 (1.09))	82.9% (3.05 (0.94))	*χ*^2^(1) = 4.60, *p* = 0.032 [72.7%] [2.86 (0.77)]
To what extent did the manual help you cope with your problems? (it helped me cope with almost all of my problems (1), it helped me cope with the majority of my problems (2) vs. it helped me very little (3), it did not help me at all (4))^a^	51.4% (2.76 (1.00))	65% (2.61 (0.97))	*χ*^2^(1) = 1.47, *p* = 0.225 [59.1%] [2.55 (0.91)]
Would you recommend the manual to a friend with similar symptoms? (definitely not (1), probably not (2) vs. probably yes (3), absolutely (4))	73.7% (3.00 (0.93))	85.7% (3.25 (0.81))	*χ*^2^(1) = 1.80, *p* = 0.179 [77.3%] [3.14 (0.99)]
How happy are you about the extent of the help you have received through using the manual? (dissatisfied (1), somewhat dissatisfied (2) vs. mostly satisfied (3), very satisfied (4))	77.1% (3.05 (1.04))	77.5% (3.07 (1.02))	*χ*^2^(1) = 0.00, *p* = 0.971 [68.2%] [2.91 (0.87)]
Did the manual help you cope with your problems more successfully? (yes, it absolutely helped me (1), yes, it helped me a little (2) vs. no, it did not help me that much (3), no, it did not help me at all (4))^a^	58.3% (2.45 (0.95))	71.8% (2.45 (1.15))	*χ*^2^(1) = 1.50, *p* = 0.221 [86.4%] [1.91 (0.87)]
How satisfied are you with the manual in general? (very satisfied (1), mostly satisfied (2) vs. somewhat unsatisfied (3), unsatisfied (4))^a^	70.3% (2.34 (0.99)	79.5% (2.39 (1.22))	*χ*^2^(1) = 0.860, *p* = 0.354 [77.3%] [2.09 (0.87)]
Would you use the manual again? (definitely not (1), probably not (2) vs. probably yes (3), yes (4))	63.2% (2.87 (1.02))	84.6% (3.45 (0.93))	*χ*^2^(1) = 4.61, *p* = 0.032 [77.3%] [3.09 (1.15)]

^a^Lower scores indicate more positive values (inverted scores)

### Imaginal retraining

Imaginal retraining is a manualized self-help intervention (10 pages with 5 figures; 3918 words). The manual starts with a short psychoeducational section that highlights well-established negative consequences of overweight and obesity in order to increase motivation to change. Next, participants are familiarized with findings using the classical approach-avoidance procedure (diagnostic and intervention [ABM] procedure). To enhance participants’ understanding of the rationale for our procedure, the psychological mechanisms that seem to underlie conventional retraining are explained. Some disadvantages of the original technique (need for access to a computer, lack of personalization, boredom) are identified as issues that our procedure aims to address.

Before conveying the technique of imaginal retraining, we describe exposure in vivo and in sensu, as the latter intervention is an ingrained part of our procedure. Participants are then instructed to imagine their preferred high-calorie foods that they want to eat less of along with their usual environment when eating these foods. Following this, they are asked to imagine a healthy, low-calorie food they also like to eat (e.g., apples). Participants are informed about the close connection between body posture, thoughts, and emotions. We illustrate that when we are in a bad mood, we tend to walk slumped over and with the corners of our mouths turned down, whereas when we feel happy, we walk with a more upright body posture and have a confident facial expression. Posture and emotion influence each other reciprocally. Thus, straightening the body leads to a slight improvement in mood, whereas adopting a bent-over posture tends to reduce well-being.

**Fig. 2:**
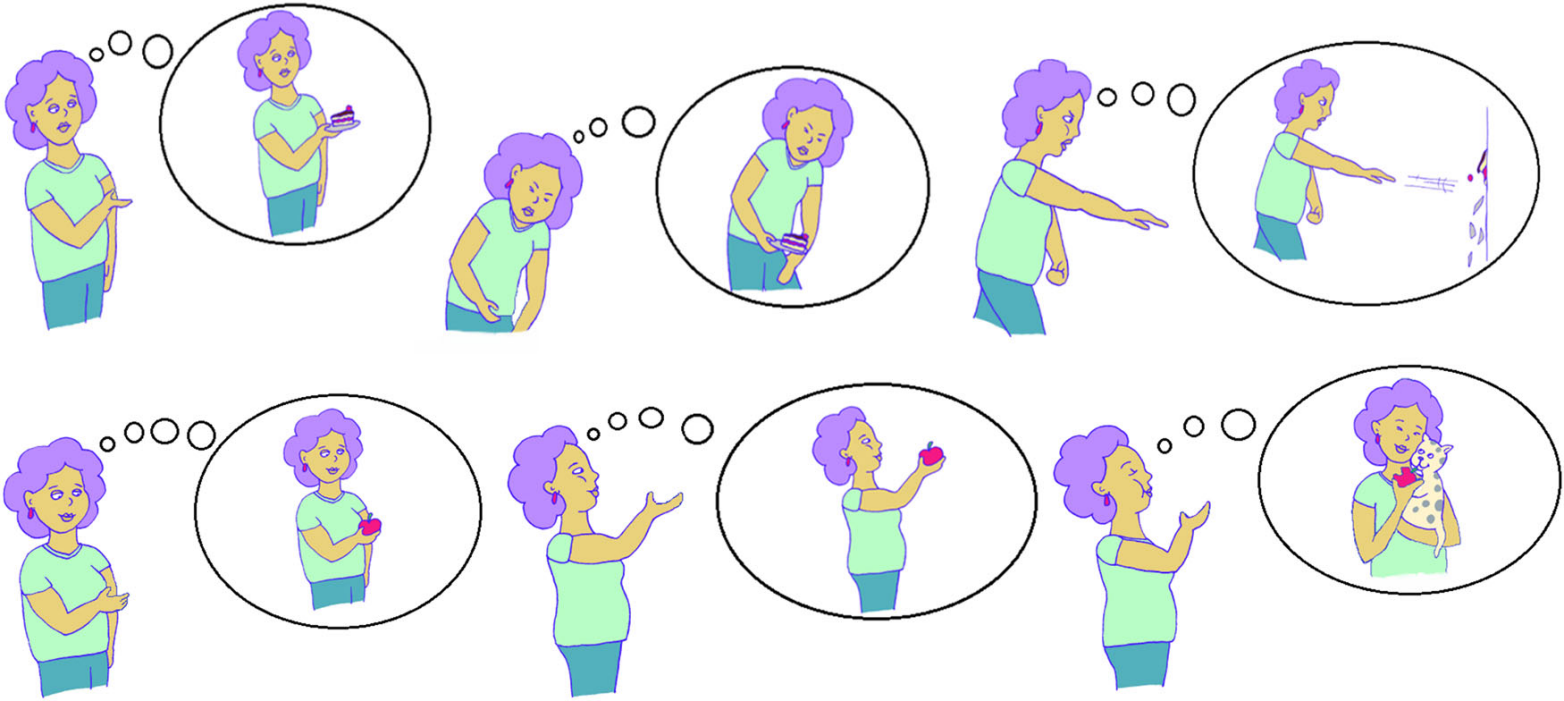
The two steps of imaginal retraining. Aversion sequence (upper panel): The individual should imagine grabbing a favorite high-calorie food, bend forward and contemplate negative thoughts, and then throwing the imagined food away from themselves (actual movement with imagined object). Approach sequence (lower panel): The individual should imagine grabbing a healthy food such as an apple, stand upright, lift the imagined food high and, if possible, couple this with other positive feelings (e.g., stroking a pet; actual movement with imagined object).

Then, imaginal retraining is taught, which consists of two sequences with two steps each. In the first part, the participant is asked to first exhale, slump forward, and round his or her shoulders. This posture should be reinforced as vividly as possible with negative thoughts (i.e., mood induction). Subsequently, participants should imagine their favorite high-calorie food and the place where they usually consume it. Participants should then push or throw the food away from themselves *in their imagination* (e.g., throwing a cake against the wall) while vigorously executing the movement *in reality*. This sequence is depicted in the manual by a series of drawings (see Fig. 2). We also advise participants to throw the imagined food onto the ground because pushing away and downward movements are both typically associated with disgust and rejection.

For the approach sequence, participants are instructed to imagine eating healthy food (e.g., an apple). They are asked to take a deep breath and stand up straight and tall as if someone were pulling them up by an imaginary string attached to the top of their head. They are then told to move the imagined healthy food or drink toward their mouth in a somewhat exaggerated, happy manner, as actors often do in advertisements, so that they are looking slightly upwards (to improve mood). At the same time, they should contemplate pleasant thoughts and images (e.g., eating an apple while stroking a pet lying against their chest). Again, drawings are used to depict this procedure (see Fig. 2). Participants are asked to perform the exercises regularly, at least twice a day. No further tips (e.g., regarding a diet or physical exercises) are provided.

After one week, half of the participants in the retraining group (the R_ER_ group) were sent specific instructions that included screenshots on programming their android or iOS smartphone to receive messages once or twice daily.

### Questionnaires

#### Primary outcome

*Visual Analog Scale (VAS) for food craving*. The mean total score of a three-item VAS scale served as the primary outcome. This scale was used in analogous trials in problem drinkers^24^ and smokers^26^. The VAS referred to the previous week. Participants had to move a bar between 0 and 100 to represent the strength of their high-calorie food craving in non-eating phases (not at all [=0] to very strong [=100]); strongest craving for high-calorie food (not at all [=0] to very strong [=100]); and frequency of craving for high-calorie food (never [=0] to always [=100]). Thus, higher scores indicated stronger craving.

#### Secondary outcomes

*Food Cravings Questionnaire-Trait-reduced (FCQ-T-R*^28^). The FCQ-T-R is a short version of the Food Cravings Questionnaire-Trait (FCQ-T^33^. The questionnaire is sensitive to change^34,35^ and is correlated with the BMI, as well as with impulsiveness and is negatively correlated with success in diets^28^. The short form consists of five subscales: lack of control over eating (five items; Lack of Control), thoughts or preoccupation with food (five items; Thoughts/Preoccupation with Food), intentions and plans to consume food (two items; Intentions to Consume), emotions before or during food craving (two items; Emotions), and cues that may trigger food craving (one item; Triggers). Following Holmes and Meule^36^, items have to be endorsed on a 5-point Likert scale. Internal consistency (*α* = 0.94) as well as test–retest reliability (*r* = 0.74) are good^28,37^. Higher scores indicate greater problems with eating behavior.

*Three-Factor Eating Questionnaire* (Fragebogen zum Essverhalten, FEV^27^). The German FEV is a multidimensional questionnaire and incorporates the English Three-Factor Eating Questionnaire (TEFQ) by Stunkard and Messick^38^, a commonly used instrument^39^. Three subscales exist. The first is cognitive restraint from eating (21 items). A high score represents spontaneous and virtually uncontrolled binge eating; a low score hints at controlled eating by means of, for example, counting calories (e.g., “I have a pretty good idea of the number of calories in common foods”; ”I consciously hold back at meals in order not to gain weight”). The subscale disinhibition is composed of 16 items. A high score shows cue-driven excessive eating behavior due to, for example, emotional turmoil and external triggers (e.g., “I usually eat too much at social occasions, like parties and picnics”; “When I feel anxious, I find myself eating”). The third scale taps hunger. A high score indicates bothersome sensations of hunger (e.g., “I am usually so hungry that I eat more than three times a day”; “Dieting is so hard for me because I just get too hungry”). For the German version^27^, high validity and internal consistency has been reported (Cronbach’s *α* = 0.74–0.87).

*Beck Depression Inventory II (BDI-II*^40^). The BDI-II contains 21 items that capture common somatic and psychological symptoms of depression. The internal consistency and test–retest reliability of the German version are good^41^.

WHO Quality of Life (WHOQOL-BREF^29^). The global item of the WHOQOL-BREF served as an index of quality of life.

#### Subjective appraisal and benefit

Participants who had at least started to read the manual were asked to fill out the Patient Satisfaction Questionnaire (German acronym ZUF-8^42^, adapted for online interventions). The scale assesses subjective appraisal of the technique (e.g., quality, satisfaction, effectiveness, and intention to use the application in the future). Tables 4 and 5 show the results of these and additional questions pertaining to treatment.

## Results

The final sample consisted of 384 women with a BMI > 25. A total of 200 participants were allocated to the control condition, while 91 were allocated to standard retraining (R_S_) and 93 to retraining with electronic reminders (R_ER_). Baseline sociodemographic and eating-relevant characteristics of the three conditions are displayed in Table 1. The participants were in their late 40s. The mean BMI corresponds to obesity, and the mean BDI-II suggests the presence of minimal to mild depressive symptoms. Approximately one-third of the sample (33.1%) had attempted one to three diets in the past. Almost one-fifth (20.5%) had attempted more than nine diets or were on a permanent diet. Approximately the same percentage had never tried a diet (20.6%).

**Table 5:** Subjective appraisal of the retraining manual, with means and standard deviations (results from problem drinkers are inside square brackets).

Item	Imaginal retraining standard (R_S_; *n* = 38)	Imaginal retraining with reminders (R_ER_; *n* = 44)	Endorsement in % (fully applies through applies a little)
I think the retraining manual is good for self-help and self-guidance.	92.1% (3.08 (0.97))	97.7% (3.02 (0.88))	*χ*^2^(1) = 1.39, *p* = 0.239 [100%] [2.91 (0.75)]
My consumption of high-calorie food has decreased because of the application of the program.	63.2% (2.13 (1.07)	79.5% (2.09 (0.77)	*χ*^2^(1) = 2.713, *p* = 0.100 [72.7%] [2.41 (1.10)]
I think the content of the manual was comprehensible.	100% (3.58 (0.64))	100% (3.52 (0.70)	n/a [100%] [3.55 (0.60)]
I think the manual was helpful.	86.8% (2.68 (1.02))	93.2% (2.91 (0.88)	*χ*^2^(1) = 0.931, *p* = 0.335 [90.9%] [2.91 (0.97)]
I was able to use the manual on a regular basis during the past six weeks.	55.3% (1.95 (0.98))	70.5% (2.25 (1.04))	*χ*^2^(1) = 2.028, *p* = 0.154 [86.4%] [2.73 (1.03)]
I had to force myself to use the manual.	76.3% (2.74 (1.18))	84.1% (2.45 (0.98))	*χ*^2^(1) = 0.785, *p* = 0.376 [68.2%] [2.00 (0.82)]
I think the manual would make more sense if it were used in combination with psychotherapy.	81.6% (2.84 (1.08))	93.2% (3.02 (0.93)	*χ*^2^(1) = 2.564, *p* = 0.109 [95.5%] [3.00 (0.82)]
The manual is not applicable to my eating behavior.	55.3% (1.95 (1.06)	52.3% (1.84 (0.99)	*χ*^2^(1) = 0.73, *p* = 0.787 [40.9%] [1.68 (0.95)]

*Note*: Scoring: 1 = not at all, 2 = a little, 3 = a lot, 4 = absolutely, n/a = not applicable

Adherence was comparable across groups (controls: 74.5%; standard imaginal retraining without electronic reminders [R_S_]: 69%; retraining with electronic reminders [R_ER_]: 73%), *χ*^2^ (2) = 0.88, *p* = 0.643. Being on a diet during the study period did not influence either craving or weight and did not interact with intervention in the two-way ANOVA with Group and Diet (yes, no) as factors (*p* > 0.2)

Of those in the two retraining conditions who agreed to rate their degree of engagement with the treatment, 5% acknowledged that they had not read the manual at all (prior study on smoking: 8.5%), whereas 26.7% reported they had read the manual at least partially but had not (yet) performed any exercises (prior study: 43.9%); 18.3% had performed the exercises once in the intervention period (prior study: 24.4%); and 17.5% had performed the exercises once a week (prior study: 24.4%). Almost one-fourth (24.2%) had performed the exercises several times a week (prior study 17.1%). Six participants (5%) had performed the exercises on a daily basis (prior study: 1.2%). Few participants (3.3%) had performed the exercises several times daily (prior study: 4.9%). Thus, approximately two-thirds of the sample (68.3%) had performed the exercises at least once during the study period.

Of the controls, 24.2% (prior study on smoking: 26.4%) self-reported eating less during the intervention period compared to 36.1% in the R_S_ and 39.4% in the R_ER_ conditions (prior study on smoking: 45.5%), *χ*^2^(2) = 6.19, *p* = 0.045. The latter rates rose to 45.9% and 46.5%, respectively, when considering only those who had performed the exercises at least once in the treatment period (prior study: 59%), *χ*^2^(1) = 11.70, *p* = 0.003.

### Utilization and dose effects

Participants in the standard R_S_ condition used the technique somewhat less often than those in the *R*_ER_ condition who were encouraged to use electronic reminders (criterion: usage multiple two times a week or more often: 24.1% vs. 40.3%), *χ*^2^(1) = 3.58, *p* = 0.059.

The frequency of performance of the technique (i.e., only read manual; performed exercises once during the study period; performed once weekly; performed several times a week; performed daily; performed several times daily) correlated with changes on certain eating-related parameters suggestive of a beneficial effect of the intervention on outcome: VAS (*r* = 0.22, *p* = 0.015), FCQ-T-R Total (*r* = 0.35, *p* < 0.001), FCQ-T-R Lack of Control (*r* = 0.37, *p* < 0.001), FCQ-T-R Thoughts/Preoccupation with Food (*r* = 0.28, *p* = 0.002), FCQ-T-R Intentions to Consume (*r* = 0.33, *p* < 0.009), FEV Cognitive Restraint (*r* = −0.24, *p* < 0.009), and FEV disinhibition (*r* = 0.23, *p* < 0.012).

### Test–retest reliability

Test-retest reliability was satisfactory to good, with the following scores (in ascending order of strength): weight (*r* = 0.98), BDI-II (*r* = 0.79), FEV Cognitive Restraint (*r* = 0.72), and FCQ-T-R Total (*r* = 0.70; FCQ-T-R subscales: *r* *=* 0.56–0.73). Reliability of FEV Disinhibition (*r* = 0.69), FEV Hunger (*r* = 0.66), the QoL global item (*r* = 0.60), and the VAS composite score (*r* = 0.49) were acceptable (all *p* < 0.001).

### Intention-to-treat and per-protocol analyses

Group differences across time were calculated using mixed ANOVAs with Group as the between-subjects and Time as the within-subject factor. The intention-to-treat (ITT; missing values were estimated using the expectation-maximization method) and the within-subject analyses results are given in Table 2. Except for quality of life, participants improved on all scores, including depression, irrespective of group allocation. The pooled imaginal retraining group displayed greater improvements than controls on four outcomes: FEV Cognitive Restraint, FEV Hunger, FCQ-T-R Total, and FCQ-T-R Lack of Control. Yet, no differences emerged on the VAS, the primary outcome. When analyses were confined to the retraining group with electronic reminders (R_ER_), which showed better adherence than the standard (R_S_) group, seven outcomes, including the primary outcome, yielded significance: VAS, FEV Cognitive Restraint, FEV Disinhibition, FCQ-T-R Total, FCQ-T-R Lack of Control, FCQ-T-R Thoughts/Preoccupation with Food, and BDI-II (results pertaining to the BDI-II should be interpreted cautiously in view of baseline differences). Effect sizes for significant results were in the small to medium range. In the subgroup in which weight was measured with a scale, the pooled retraining group showed a greater weight loss at statistical trend level, which achieved significance when considering the R_ER_ group only (see Table 2).

Results were corroborated by the per-protocol (PP) analyses, considering only those participants who had used the technique at least once in the treatment period. Again, participants improved overall at a medium to large effect size except for quality of life. The pooled retraining group was superior to the control group on seven out of 12 outcomes: VAS, FEV Cognitive Restraint, FEV Hunger, FCQ-T-R Total, FCQ-T-R Lack of Control, FCQ-T-R Intentions to Consume, and FCQ-T-R Triggers. At statistical trend level, weight loss was more marked in the pooled retraining group when considering those who endorsed that they had measured their weight using a scale at both baseline and reassessment. When constricting analyses to the R_ER_ group, this outcome achieved significance at a medium effect size. Again, for seven outcomes the R_ER_ group had better results than controls; two results bordered on significance and three were nonsignificant.

The two retraining subgroups performed almost equally except on the QoL (*p* = 0.016, *η*_p_^2^ = 0.070) and FCQ-T-R Emotions (*p* = 0.042, *η*_p_^2^ = 0.051), where the R_ER_ displayed grater improvement. The paired comparisons indicate that group differences were due to improvement in the experimental conditions and not worsening in the control condition. In fact, controls improved on most outcomes across time.

### Completers vs. non-completers

Compared to non-completers, completers were older (*p* = 0.017), more often endorsed that they had attempted more than 15 diets (*p* < 0.001), had somewhat higher treatment expectancy (*p* = 0.044), and scored higher on FEV Cognitive Restraint (*p* = 0.039).

### Subjective appraisal

Tables 4 and 5 display the subjective appraisals of the participants in the two retraining groups who had performed the exercises at least once. Appraisals were better for the group with electronic reminders on the following parameters: whether they had received the expected treatment and whether participants would use the manual again. At trend level, the electronic reminder group more often endorsed that their consumption of high-calorie food had decreased because of their use of the program (79.5% vs. 63.2%). More than 80% of the participants endorsed the following statements: good or excellent quality of the manual, would recommend the manual to a friend (R_ER_ only), good for self-help, comprehensible, the manual would have made more sense if it were used in combination with psychotherapy, I had to force myself to use the manual (R_ER_ only), expected type of treatment received (R_ER_ only), and helpful and would use manual again (R_ER_ only). Between 70% and 79.9% endorsed the following items: I had to force myself to use the manual (R_S_ only), helped me cope with problems more successfully (R_ER_ only), satisfied with the manual in general, happy about the extent of help received from the manual, consumption of high-calorie food decreased because of the program (R_ER_ only), able to use the manual on a regular basis (R_ER_ only), and would recommend the manual to a friend (R_S_ only). Between 60% and 69.9% endorsed the following statements: would use manual again (R_S_), consumption of high-calorie food decreased because of the program (R_S_ only), helped me cope with problems (R_ER_ only), and expected type of treatment received (R_S_ only).

For use as a benchmark, the figures from our forerunner trial on imaginal retraining in problem drinkers are set in square brackets in Tables 4 and 5. We comment on the differences to the benchmark if they exceed 10%. More participants in the current trial (R_ER_: 52.3%, R_S_: 55.3%) endorsed that the manual was not applicable to their problem behavior than in the alcohol study (40.9%). In particular, the participants in the R_S_ condition endorsed less often that they were able to use the manual on a regular basis during the previous 6 weeks (55.3% vs. 86.4% in the prior study). Again, less participants in the current trial (R_ER_: 71.8%, R_S_: 58.3%) endorsed that the manual helped them cope with their problems more successfully relative to the alcohol study (86.4%). Yet, their happiness about the help they received was markedly higher.

## Discussion

Individuals intending to lose weight usually either start a diet aimed at the reduction of calorie intake or initiate/enhance recreational activities (e.g., sports) aimed at burning additional calories. Such measures necessitate effort and discipline and are often short-lived. Once they end, the weight often increases^43,44^, sometimes above baseline due to hormonal changes (i.e., the yo–yo effect^45^).

We borrowed the present technique from the computerized ABM paradigm, which has frequently been administered in studies related to eating^20,46^, but we pursued another route. We expected the intake of and craving for high-calorie food to be reduced by a simple, quick technique that dampens the craving/addiction-like properties of conditioned high-calorie food stimuli. Participants learn to couple the image of their favorite unhealthy/high-calorie food with a mildly aversive emotion (mood induction) and a push movement, similar to classical ABM but with no need for technical equipment^24^. This imaginal variant of retraining has been successfully tested in problem drinkers^24^ and smokers^26^, and the technique reduced craving and also actual consumption. The present study transposed the approach to food.

Unique to this study, two retraining conditions were set up to test whether adherence could be improved by reminders. Similar to the forerunner studies, one group, the standard retraining (R_S_) group, received the manual with instructions to read through the manual and to perform the exercises on a daily basis. The other group (R_ER)_ received the same manual but was also sent an email after one week with specific instructions on how to use the timer on their smartphone in order to give them daily reminders to perform the exercises. This condition was implemented following evidence that daily smartphone notifications to perform simple exercises foster reduction of target symptoms^47^. Indeed, the latter group performed the exercises more regularly, and this frequency in turn was associated with improvement on several outcomes at a small to medium effect size. Accordingly, overall effects were larger for the R_ER_ group than for the other retraining group. Both ITT and PP analyses indicate that this subgroup benefited from the intervention in terms of craving and also psychopathological symptoms (depression). For the small subgroup who had measured their weight before and after the study period with a scale, weight loss was more than 3 kg, which represented a medium effect size relative to controls. The pooled retraining group showed a significant effect on craving in the PP but not the ITT analyses. Effects on five outcomes, however, were significant for both kinds of analyses: FCQ-T-R Lack of Control, FCQ-T-R Triggers, FCQ-T-R Total, FEV Cognitive Restraint, and FEV Hunger.

Individuals in both retraining groups reported eating less than controls during the intervention period (R_ER_: 39.4%; R_S_: 36.1% vs. Control: 24.2%), and the majority would use the training again and recommend it to others. Adherence was larger than in smokers. Satisfaction was less for a number of the outcomes relative to the study on alcohol (e.g., able to use the manual on a regular basis, helped me cope with problems). Overall, R_ER_ received more favorable assessments than R_S_, which reached significance for two outcomes. The appraisal of the R_ER_ technique in individuals with overweight was numerically better on 14 of the items relative to R_S_ and on 11 of the outcomes compared to the treatment manual for problem drinkers.

Effects for participants in the current study were significant on core outcomes but somewhat weaker than for smokers and drinkers. At this point, we can only speculate about possible reasons. First, smaller effects were likely due to unexpected improvements in the wait-list control group. Controls also reduced weight as well as craving and improved on other outcomes. Second, the range of possible stimuli is narrower in smokers and drinkers, who often have a typical “drug of choice” (e.g., bottle of red wine; certain brand of cigarette). For eating, stimulus control is poor because even the same type of food (e.g., cake, cookies) can have many different shapes or colors. This assumption can be tested, however. For example, we would expect a greater reduction of weight and craving in overweight persons who imagine a wide range of unhealthy foods they like to eat during the retraining compared to those who imagined only a few unhealthy foods they like to eat (e.g., chips, pizza, and soda). Lastly, the imaginal retraining manual for drinkers gave additional recommendations that might have augmented the effect. However, no such tips were provided to smokers, so this cannot explain the efficacy gap in the study of smokers vs. the current study of people with overweight or obesity.

Motivation to engage the manual was higher than in smokers, but still 31.7% of the participants did not perform the exercises. Even though the technique is simple, other ways of presentation should be tested, such as video clips via social media demonstrating how to perform the exercises. The technique contains many elements. Future research should elucidate the differential effectiveness of its various elements and whether certain elements are dispensable, such as negative mood induction and the positive counter-sequence. Also, it would be helpful to know whether adding further behavioral tips to the manual or encouraging a special diet would enhance the effect.

The study is subject to a number of limitations. First, we do not know whether the effects are stable over time, and we have not yet tested long-term adherence to the technique. Relatedly, at this point we can only speculate which aspects of the eating behavior had changed (e.g., overall reduction or eating less unhealthy vs. eating more healthy food). Second, we relied on self-reports, which are prone to the effects of social desirability; future trials should monitor participants under more controlled conditions with objective assessments, including weighing with a scale^2^. As in our forerunner trials, we refrained from implementing a sham condition for ethical reasons. Third, the efficacy of the technique needs to be explored in males as well, who have a higher prevalence of obesity, which seems to be more strongly related to alcohol consumption than in females^48^. Fourth, for exploratory purposes we did not control for multiple comparisons, which would have rendered many secondary outcomes non-significant. Fifth, future studies should explore motifs for non-adherence more rigorously (e.g., forgetting, inefficacy despite attempt to use it, no confidence in the rationale). Sixth, subsequent trials should test the present approach against an active control which may attenuate placebo and expectancy effects. Finally, whether the technique reduced craving and approach behavior as assessed by implicit measures has yet to be studied.

## Conclusions

In individuals with overweight (BMI > 25), many of whom met criteria for obesity, imaginal retraining significantly reduced craving for high-calorie food over a period of 6 weeks. A small to medium correlation emerged between utilization frequency and outcome. Treatment effects were larger in the subgroup encouraged to use electronic reminders than in the pooled retraining group. The long-term effects of the technique in terms of acceptance, feasibility, and effectiveness need to be tested as well as alternative ways of presenting the technique (e.g., video clips) and the long-term efficacy.

## Footnotes

1 Craving for food relates to the strong desire to consume a specific food and is different from normal hunger; craving for food is particularly prevalent for unhealthful food that is high in calories from fat or sugar.

2 We would like to thank one reviewer who suggested that those who had lost weight were perhaps more likely to recognize this and as a result both measured and reported their weight.
